# Mesenchymal Stem Cells and Their Extracellular Vesicles: A Potential Game Changer for the COVID-19 Crisis

**DOI:** 10.3389/fcell.2020.587866

**Published:** 2020-09-30

**Authors:** Dina H. Kassem, Mohamed M. Kamal

**Affiliations:** ^1^Department of Biochemistry, Faculty of Pharmacy, Ain Shams University, Cairo, Egypt; ^2^Department of Pharmacology and Biochemistry, Faculty of Pharmacy, The British University in Egypt (BUE), Cairo, Egypt; ^3^The Centre for Drug Research and Development (CDRD), Faculty of Pharmacy, The British University in Egypt (BUE), Cairo, Egypt

**Keywords:** COVID-19, cytokine storm, extracellular vesicles, exosomes, immunomodulation, mesenchymal stem cells, SARS-CoV-2, corona virus

## Abstract

Corona virus disease 2019 (COVID-19) is a global public health crisis. The high infectivity of the disease even from non-symptomatic infected patients, together with the lack of a definitive cure or preventive measures are all responsible for disease outbreak. The severity of COVID-19 seems to be mostly dependent on the patients’ own immune response. The over-activation of the immune system in an attempt to kill the virus, can cause a “cytokine storm” which in turn can induce acute respiratory distress syndrome (ARDS), as well as multi-organ damage, and ultimately may lead to death. Thus, harnessing the immunomodulatory properties of mesenchymal stem cells (MSCs) to ameliorate that cytokine-storm can indeed provide a golden key for the treatment of COVID-19 patients, especially severe cases. In fact, MSCs transplantation can improve the overall outcome of COVID-19 patients via multiple mechanisms; first through their immunomodulatory effects which will help to regulate the infected patient inflammatory response, second via promoting tissue-repair and regeneration, and third through their antifibrotic effects. All these mechanisms will interplay and intervene together to enhance lung-repair and protect various organs from any damage resulting from exaggerated immune-response. A therapeutic modality which provides all these mechanisms undoubtedly hold a strong potential to help COVID-19 patients even those with the worst condition to hopefully survive and recover.

## Introduction

Corona virus disease-2019 (COVID-19) is a devastating growing pandemic. As of 6th September 2020, the World health Organization (WHO) has reported 26,763,217 confirmed cases and 876,616 confirmed deaths resulting from COVID-19 globally (World Health Organization [WHO], 2020a). The severe acute respiratory syndrome corona virus-2 (SARS-CoV-2) is the inducing initiating factor which causes COVID-19 (Gorbalenya et al., 2020). The viral infection occurs mainly due to aerosol particles resulting from talking and breathing of an infected person, which pose an infection-threat if they are transferred to a nearby person (Meselson, 2020). Moreover, if these particles settle on surfaces, they represent a contact hazard since the virus remains infective/viable for several hours (at least) depending on the nature of the surface (van Doremalen et al., 2020). Unfortunately, SARS-CoV-2 is characterized by being highly infectious, and in the same time capable of evading the human immune-response; both features may at least partially explain the COVID-19 outbreak (Shang et al., 2020a). It is important to point here that SARS-CoV-2 enters the human cells via angiotensin-converting enzyme 2 (ACE2) receptor; a process in which human proteases act as entry-activators (Shang et al., 2020a,b). Moreover, Shin and coworkers recently highlighted how SARS-CoV2 utilizes a papain-like protease to cleave ISG15 (an ubiquitin-like protein) from interferon responsive factor 3 (IRF3). This attenuates the type I interferon responses, and helps the virus to evade immune response (Shin et al., 2020).

### COVID-19 Clinical Presentation and Complications

The clinical presentation of COVID-19 ranges from asymptomatic presentation to severe respiratory-tract infection in the form of pneumonia, which can be fatal (Deshmukh et al., 2020; Tay et al., 2020). In fact, not all patients infected with SARS-CoV-2 develop severe respiratory illness (Shi et al., 2020). Most of the COVID-19 patients will experience relatively mild symptoms such as dry cough, fatigue, fever, and myalgia (Li et al., 2020). However, severe COVID-19 cases can progress to acute respiratory distress syndrome (ARDS) with significant hypoxia, as well as multi-organ involvement such as renal-failure, heart-failure, liver-damage, and shock which precipitates death (Zaim et al., 2020). High incidence of venous thromboembolic complications and abnormal coagulation variables have also been noted during the investigations of COVID-19 autopsies (Deshpande, 2020).

It’s important to point here that both mild and severe forms of COVID-19 have been reported to be associated with changes in circulatory cytokine levels such as IL-6, IL-1β, and others (Wang J. et al., 2020). Moreover, severe complications of COVID-19 are associated with the hyper-production of cytokines, known as “cytokine storm” (Shi et al., 2020; Wang J. et al., 2020). Thus, the immune-response is undoubtedly one of the key determinants not only of the susceptibility but also of the disease-severity. On one hand, weak immune system can increase the risk of SARS-CoV-2 infection. While on the other hand, hyper-inflammatory response to the viral-infection can be responsible for the disease severe complications (Shi et al., 2020; Zaim et al., 2020). Furthermore, accumulating evidence suggests that cytokine-storm is the main predictor of mortality, and that identification and treatment of hyper-inflammation is recommended to reduce the rising mortality (Mehta et al., 2020). It’s also important to highlight here that elderly people (over 60 years), and those with health conditions such as heart or lung disease, diabetes, or conditions that might affect their immune system are at most risk for developing severe COVID-19 (World Health Organization [WHO], 2020b).

### Immunopathology of COVID-19

Severe acute respiratory syndrome corona virus-2 or COVID-19 is a member of coronoviridae virus family. It is an enveloped single RNA strand virus with a spherical virion and spiked glycoprotein, hence the popular name of corona virus of this family (Allegra et al., 2020). After entering the lungs through respiration, SARS-CoV-2 stimulates the activity of immune cells and increase cytokine production. As any other RNA virus, SARS-CoV-2 can be identified through toll like receptor (TLR) which stimulates IFN-γ production and activates CD8 + T cells, natural killer cells and macrophages (Nelemans and Kikkert, 2019). The infected cells then present the viral antigen as portions of surface antigen-MHC-I complexes which activates cytotoxic T-lymphocytes (Baruah and Bose, 2020). SARS-CoV and MERS-CoV infections are characterized by fast and robust initial virus replication with late IFN generation, resulting in disproportionate inflammatory host responses provoking grave lung alterations (Kindler and Thiel, 2016). In the fight between the virus and the human body, the immunity of the subjects reduces, and the virus virulence augments. This causes edema and congestion of the lung, thickening of the interstitial tissue, and augmented exudation in the alveolar space able to cause respiratory failure (Han et al., 2020).

As for SARS-CoV-2 interactions with the immune cells, several studies showed that T-cells, B-cells and NK cells were significantly reduced in patients. Besides, T-cytotoxic and even T-helper cells were all below normal range (Liu et al., 2020; Qin et al., 2020). In addition, cytokines play important role in the immunopathology of the viral infection. Many cytokines were found to be elevated in SARS-CoV-2 infected subjects including IL-1β, IFN-γ, monocyte chemoattractant protein-1 (MCP-1), IL-17, IL-2R, and IL-6 (Huang et al., 2020; Zhang W. et al., 2020). Not only are these cytokines elevated, but also they are correlated with the disease severity (Chen L. et al., 2020). Of special interest of these cytokines, several reports have analyzed IL-6 plasma concentration. Most studies tend to consider IL-6 elevated levels as a negative factor in SARS-CoV-2 pneumonia. IL-6 can inhibit CD8 + cytotoxic T-cells by reducing IFN-γ and it can block antiviral response during the cytokine storm (Velazquez-Salinas et al., 2019). That’s the reason why IL-6-R antagonist tocilizumab has found its way to many of the current protocols used to control COVID-19 mediated pneumonia. However, IL-6 also plays an essential role in lung repair responses after viral injury, suggesting that the timing of administration of anti-IL6R could interfere with correct tissue remodeling (Okabayashi et al., 2006).

Actually, usually in the beginning of the SARS-CoV-2 infection, the patients are presented with mild symptoms, however, the clinical condition is worsened unexpectedly in later stage of the disease due to what is called “cytokine storm” causing ARDS and multiple organ failure (Teijaro et al., 2014). Hence, controlling this cytokine storm is essential for treating the COVID-19 ARDS. Beside the previously described cytokines, studies showed that cytokine storm is related to T-helper-17 (Th17) responses. IL-1β and TNF-α both increase Th17 responses and vascular permeability and leakage. Th17 cells themselves generate IL-17, IL-21, IL-22, and GM-CSF. IL-17 exerts pro-inflammatory actions by promoting the production of inflammatory cytokines such as IL-1β, IL-6, G-CSF, TNF-α, and chemokines such as IL-8, IP10, KC, MIP2A, MIP3A capable of recruiting more immune cells, and matrix metalloproteinases that contribute to tissue injury and remodeling. IL-17 and TNF-α are able to increase the production of mucins, serum amyloid A, fibrinogen, and anti-apoptotic proteins (Zenewicz, 2018). This huge production of cytokines from uninhibited immune system is the key element that determines the severity of the symptoms and the mortality rate in SARS-CoV2 infection (Henderson et al., 2020). Due to the action of pro-inflammatory proteins, vascular permeability increases and a great amount of fluid enters the alveoli, causing dyspnea and respiratory failure (Leiva-Juarez et al., 2018). The therapeutic approaches used in the treatment of the various cytokine release syndromes (CRSs) could be useful for the cytokine storm of SARS-CoV-2 infection (Allegra et al., 2020).

It is not clear why some patients may develop cytokine storm while others not. However, several studies have addressed key players of this cytokine storm. Several of these studies reported elevated levels of IL-6 as an essential player in the development of such cytokine storm (Gao Y. et al., 2020; Ruan et al., 2020). Another study analyzing data from 21 patients in China reported increased levels of IL-10, IL-6, and TNF-α in severe cases (Chen G. et al., 2020). This indicates that assessment of IL-6 should be considered as a determinant or predictive marker for development of such cytokine storm which may help to prevent adverse consequences in severely ill patients.

It’s worth noting here that another immune response molecular pattern that haven’t been extensively explored in cases of COVID-19 is the damage associated molecular pattern, DAMP for short and their counteracting molecules or suppressants, SAMP for short (Land, 2020). These are endogenous molecules such as high mobility group box 1 (HMGB1), S100 proteins, nuclear and mitochondrial DNA (nDNA and mtDNA), and extracellular histones that can be released or secreted by death stimuli or cytokines and can further stimulate the immune response (Tang et al., 2012). Among these, HMGB1 is a potential target of SARS (Chen et al., 2004). It can be easily assumed that HMGB1 can have same role in COVID-19 especially that some drugs that target HMGB1 such as chloroquine, can prevent septic shock through inhibition of HMGB1 (Yang et al., 2013), inhibit SARS-CoV-2 replication/infection *in vitro* (Wang M. et al., 2020) and meanwhile proved some clinical efficacy in some trials (Gao J. et al., 2020). Another interesting target is Transmembrane protein 173, TMEM173, which is one of the intracellular immune regulator to pattern recognition receptors (PRR) activation during infection and tissue injury (Tang et al., 2020). However, these DAMPs and SAMPs worth further investigation, not only for a search of specific molecular pattern that would help to better diagnose COVID-19, but also for mining of additional therapeutic targets that can either control the hyper-inflammatory response or halt the viral replication.

### Immunomodulatory Therapeutic Strategies for COVID-19

The hyper-inflammation state or “cytokine release storm” induced by COVID-19 is the basis of how immune-modulatory and/or immunosuppressive-drugs found their way to the different treatment-protocols used for severe cases of COVID-19. One of these drugs is tocilizumab, an IgG1 subclass antibody that inhibits IL-6 receptor (World Health Organization [WHO], 2020b). It is approved for treating inflammatory/autoimmune diseases such as rheumatoid arthritis (Shin et al., 2020). Despite the fact that immunosuppressive-drugs are generally not recommended during viral infections (Russell et al., 2020), tocilizumab has been tested in COVID-19 patients with elevated IL-6 levels, and exhibited several beneficial effects (Witkowski et al., 2016; Moll et al., 2019). Currently, registered multicenter randomized controlled phase-II clinical trials to further assess its efficacy and safety for COVID-19 are recruiting patients (NCT04335071 and ChiCTR2000029765) (ClinicalTrials.gov, 2020; Chinese Clinical Trial Registry, 2020). Recently, tocilizumab was added to remdesivir for hospitalized-patients with severe COVID-19-induced pneumonia in REMDACTA study (NCT04409262) (ClinicalTrials.gov, 2020). By the way, the only evidence-based COVID-19 available treatment is remdesivir which is a viral RNA-polymerase inhibitor that shortens time to hospital discharge (Beigel et al., 2020).

Another popular immunosuppressive-drug whose association with decreased-mortality was considered a “breakthrough” in our battle with COVID-19 is dexamethasone. Severe COVID-19 patients given 6 mg dexamethasone once daily had 8–26% lower mortality compared to patients with standard care in RECOVERY study (Group, 2020; Mahase, 2020). Although this study raised enormous enthusiasm about having an affordable-drug for COVID-19, however, some concerns were also raised. Generally, corticosteroids have wide effects on both innate and adaptive-immunity and the United States Center for Disease Control and Prevention (CDC) recommends against the use of corticosteroids in patients with corona-virus infection (Arabi et al., 2018). In addition, the RECOVERY results recommend the use of dexamethasone in patients with severe respiratory-distress syndrome and not out-patients (Group, 2020). However, there exists a gap of evidence in the study remaining to be fulfilled including the long term effect of dexamethasone, its effect in old-aged patients, the timing, the dose and even the type of corticosteroid needs further investigations (Johnson and Vinetz, 2020). In the middle of this feverish race to develop new treatment-protocols, repositioning old drugs to treat COVID-19 or even achieve a radical solution for this pandemic by developing a COVID-19 vaccine, mesenchymal stem cells (MSCs) appear at the end of the tunnel as a potential treatment which deserve to be considered carefully not only for this COVID-19 crisis but also for other viral infections that may occur in the future.

### Mesenchymal Stem Cells Therapy for Pulmonary Diseases

The main fascination about MSCs lies in their reported potential to exert protective and reparative effects on an amazingly wide-spectrum of tissue injury (Pittenger et al., 2019). This is further reinforced by their ease of isolation and large *ex vivo* expansion capacity, as well as demonstrated multipotency and immunomodulatory activities (Gao et al., 2016). MSCs can be isolated from various sources such as; bone-marrow, adipose-tissue, as well as umbilical-cord Wharton’s jelly (WJ-MSCs) (Pittenger et al., 2019). However, several reports showed that MSCs derived from different tissues have different transcriptomic-profile, as well as varying in-context biological/therapeutic properties (Fong et al., 2011; El Omar et al., 2014; Barrett et al., 2018).

One of the universal properties of MSCs regardless their source or type is their immune-modulatory effects. These immune-modulatory effects are mediated through both paracrine and direct cell-to-cell contact (Ma et al., 2014). MSCs can actually affect all types of immune cells including T-lymphocytes, natural killer cells and dendritic cells (Weiss and Dahlke, 2019). Additionally, MSCs secrete a plethora of cytokines which represent the corner-stone for their immune-modulatory actions (Weiss et al., 2008). These immunomodulatory-properties enabled MSCs to find their way toward inflammatory diseases such as graft-versus-host-disease (GVHD) (Zhao et al., 2019). Likewise, these properties are the basis why MSCs can find its way in the treatment of COVID-19, specifically its ARDS.

Actually this is not the first time we turn our heads toward MSCs as a potential therapy for pulmonary lung-diseases. Over the past years, several reports highlighted the therapeutic efficacy of MSCs for experimental models of lung-diseases such as ARDS (Matthay, 2015), and chronic obstructive pulmonary disease (COPD) (Cruz and Rocco, 2020). We think two major properties of MSCs nominate them as a potential therapeutic modality for lung-diseases such as COVID-19-induced pneumonia. First, MSCs have shown high degree of tropism/homing to lungs, after their intravenous application (the most common route of cell therapy application); infused MSCs were detected in the lungs of patients within 30 min (Savukinas et al., 2016; Armitage et al., 2018). In fact, high percentage of cells lodge in the lungs (Yen et al., 2020), a phenomenon that is further increased with inflammation (Ortiz et al., 2003). These cells are cleared within 24–48 h with delayed clearance during inflammatory-conditions (Armitage et al., 2018).

Second, and more importantly, while lodging in the lung, these cells are able to secrete a wide variety of soluble cytokines, angiogenic factors and extracellular-vesicles (EVs) that can efficiently ameliorate this inflammatory-condition (Khoury et al., 2020). In fact, preclinical studies proved that MSCs not only have anti-inflammatory effects but also antifibrotic, angiogenic and microbicidal effects in chronic lung-diseases (Cruz and Rocco, 2020). Moreover, MSCs-derived micro-vesicles were reported to repair the endothelial cells of cultured injured human lungs via delivery of angiopoietin-1 to those cells; which helped to restore protein permeability (Hu et al., 2018). As for their anti-microbial activity, this is mediated through secretion of antimicrobial peptides/proteins such as hepcidin, β-defensin-2, cathelicidin-LL-37, and lipocalin-2 (Alcayaga-Miranda et al., 2017). Accordingly, MSCs are strongly suggested to provide a novel effective therapeutic modality for inflammatory lung-disease (Yen et al., 2020).

Interestingly, MSCs have also been suggested to shape their immunomodulatory actions according to the micro-environment they encounter (Rolandsson Enes and Weiss, 2020). Such notion, at least partially, explains their therapeutic efficacy in various inflammatory-disease conditions despite their totally different context and background. Furthermore, these adaptive immunomodulatory properties of MSCs could provide a particular therapeutic benefit for ARDS being an acute multi-faceted heterogeneous disorder with several differences in host environment (Rolandsson Enes and Weiss, 2020). Not only that, but also provides an advantage for MSCs compared to inhibitors for individual pro-inflammatory cytokines, or recombinant anti-inflammatory ones. Nevertheless, it’s noteworthy that despite the encouraging results from pre-clinical studies, and also the reported safety of MSCs in several phase-I clinical studies, the clinical evidence for efficacy in patients is still evolving (Rolandsson Enes and Weiss, 2020).

### Mesenchymal Stem Cells Therapy for COVID-19 ARDS

Given all these beneficial immune-modulatory actions of MSCs in lung-disease, and the COVID-19-induced “cytokine storm,” MSCs can be easily considered a potential therapy for COVID-19 ARDS. Actually, MSCs can act through several axes in combating COVID-19 induced lung injury. First, MSCs can prevent the huge release of the cytokines which activate the immune system of the patients, thereby alleviating the “cytokine storm” associated with COVID-19 infection and considered the main cause of ARDS in these patients (Golchin et al., 2020; Mehta et al., 2020). Interestingly, the pattern of anti-inflammatory mediators released are specific to the inflammatory lung environment. For example, RNA viruses like COVID-19 activates TLR3 leading to MSCs activation (Waterman et al., 2010). In addition, MSCs were found to suppress the inflammation induced lung injury through their immune modulatory properties. MSCs produce prostaglandin-E2 and IL-10 which suppress macrophages, reduce production of inflammatory cytokines and reduce neutrophils recruitment to the lung (Devaney et al., 2015; Taghavi-Farahabadi et al., 2020).

Second, MSCs have also tissue repair potential and can repair the lung tissues especially type II alveolar cells. MSCs have the ability to secrete angiopoietin-1 (Ang-1) and keratinocyte growth factor (KGF) which contribute to the restoration of alveolar–capillary barriers disrupted as part of ARDS pathogenesis (Lee et al., 2013; Matthay, 2015). In addition, MSCs can restore the epithelial protein permeability by secretion of angiopoietin-1 (Harrell et al., 2019). Hereby, MSCs not only can prevent hyper-inflammation state but also can induce endogenous lung-repair (Golchin et al., 2020).

These mechanisms were completely demonstrated in 2 very recent phase-1 clinical trials. The first one was on seven patients with single dose of MSCs lacking ACE-2 (Leng et al., 2020). The symptoms of all the patients and pulmonary functions improved 2 days after transplantation and no adverse effects were seen 14 days. Peripheral lymphocytes were increased, C-reactive protein was decreased, and natural killer cells disappeared 3–6 days post-transplantation. In addition, TNF-α level was declined while IL-10, an inhibitory cytokine, was increased. Interestingly, the chest computed tomography (CT) scan on the ninth day after MSC transplantation revealed that ground-glass opacity and inflammation of pneumonia were significantly decreased in MSCs group.

Another more recent clinical report by Meng et al., 2020, who explored transplantation of 3 doses of UC-MSCs at 3 days interval in nine patients as compared to another nine placebo receiving patients, can prove both efficacy and safety of UC-MSCs treatment in critical-ill COVID-19 patients. Interestingly, MSCs receiving patients showed lower IL-6 levels than placebo receiving patients and the authors pointed out that MSCs showed the most benefit with patients exhibiting hyper inflammation status (Meng et al., 2020). These 2 clinical trials although proved efficacy and safety of the MSCs treatment for COVID-19, however, phase 2/3 clinical trials, multi-centered and on a greater number of patients are mandatory to confirm the above promising results.

It’s important to point here that a typical immune-response to viral infection is interferon-gamma (IFN-γ), which represents an important cross-talk between COVID-19 and MSCs. An unpublished data from Italy showed that COVID-19 patients may have elevated levels of IFN-γ, which may affect MSCs therapy (Massimo Dominici, University Hospital of Modena and Reggio Emilia, Modena, Italy) (Khoury et al., 2020). IFN-γ acts as an activator of MSCs to exert their immunomodulatory actions. These activated-MSCs may suppress T-lymphocyte activation/proliferation for viral infection control (Malcherek et al., 2014). Also, UC-MSCs inhibit the cytotoxicity of specific T-cells against H1N1 influenza *in vitro* leading to prolonged infections (Liu et al., 2015). These issues highlight the importance of maintaining delicate balance to fully use the immune-suppressive properties of MSCs to prevent the cytokine-storm, meanwhile, maintain the whole body defense mechanisms toward the viral load.

Another important issue in the cross-talk between MSCs and COVID-19 is the expression of angiotensin converting enzyme-2 (ACE-2) receptor. This is the main host cell receptor for the SARS-CoV-2 entry (Perrotta et al., 2020). Also, this explains the entry of the virus into the respiratory epithelial cells as this receptor is highly expressed in type II lung alveolar cells (Xu et al., 2020; Zou et al., 2020). Nevertheless, it is important to point here that regarding definite reports highlighting which cells could be infected with SARS-CoV-2, and which cells could not be infected with the virus, such data is still emerging, and far from complete elucidation. Another study has shown that the cellular protease TMPRSS2 is also required to allow the entry of coronavirus into host cells (Hoffmann et al., 2020). It is important for MSCs to be free from SARS-CoV-2 viral infection to exert its actions properly. This was demonstrated in a recent study in China which showed that ACE^–^ MSCs, which are free of viral infection, can ameliorate COVID-19 pneumonia (Leng et al., 2020). This raises the suggestion that the expression of ACE2-receptor and TMPRSS2 should be evaluated in different types of MSCs to assess the suitability of its use with COVID-19 before clinical application. To the best of our knowledge, there are no data regarding the possibility of the infection of MSCs with COVID-19, which indeed requires further investigations.

### Mesenchymal Stem Cells Paracrine Signaling and Extracellular-Vesicles

Apart from the cross-talk between MSCs, COVID-19 and immune-cells, generally, a growing body of research has revealed that therapeutic effects of MSCs occur largely via paracrine signaling which can bypass this direct contact among MSCs and the virus (Zhang et al., 2015; Taghdiri et al., 2018). In many cases, exosomes/extracellular-vesicles (EVs) secreted by MSCs have been reported to mediate such beneficial effects (Newton et al., 2017). Exosomes/EVs act as shuttles transferring specific cargos of mRNA, non-coding RNAs, as well as proteins; resembling a message in a bottle. Accordingly, they can reprogram recipient cells due to their active molecular cargo, thus regarded as “signalosomes” for controlling fundamental cellular functions (El Andaloussi et al., 2013; Bjorge et al., 2018).

It’s noteworthy that exosomes sparked great interest as a possible cell-free alternative to current stem cell therapies. Like their parent cells, exosomes have been reported to stimulate cellular regeneration, functional recovery in various pathological conditions (Derkus et al., 2017). Moreover, recently MSC-derived EVs have been reported to suppress fibrosis by preventing differentiation of fibroblasts into myofibroblasts (Basalova et al., 2020).

When considering the therapeutic efficacy of MSCs-derived EVs compared to their parent cells for lung-diseases, that issue is a bit controversial. On one hand, an interesting pre-clinical study reported that MSCs are generally more effective than their EVs at reducing lung injury in ARDS (Silva et al., 2019). On the other hand, several pre-clinical reports highlighted the therapeutic efficacy of MSCs-derived secretome/EVs for various lung-diseases, through reduction of pro-inflammatory cytokines’ levels, and restoration of lung architecture. Collectively, these findings encouraged researchers nowadays to consider how to utilize MSCs-secretome to develop a high quality, safe and effective medicinal product (Bari et al., 2019).

### Clinical Trials for MSCs and MSCs-Derived EVs for COVID-19

Currently, about 66 clinical trials are registered in the public clinical trial database to investigate the therapeutic safety/efficacy of MSCs for COVID-19 (Accessed on 6th September, 2020) (ClinicalTrials.gov, 2020). Most of these trials are applying allogenic intra-venous route of cell-therapy, with MSCs derived mostly from umbilical-cord, adipose-tissue or bone-marrow. Currently, two registered studies are investigating therapeutic efficacy of EVs derived from MSCs via the intra-nasal route (ClinicalTrials.gov, 2020). Being a global health crisis, these trials are originating from several countries including China, the United States, Canada, Jordan, as well as, several countries in Europe such as Spain, and Germany. These various clinical trials investigating MSCs for COVID-19 are reviewed elsewhere in detail (Golchin et al., 2020; Rajarshi et al., 2020; Yen et al., 2020).

Generally, the allogenic transplantation of MSCs is preferred rather than the autologous one in case of COVID-19. This is primarily due to the rapid deterioration of COVID-19, especially in severe cases, in such a way that there will probably be no time for autologous harvesting followed by 2–3 weeks of MSCs expansion in culture (Yen et al., 2020). It’s noteworthy here that in support of allogenic MSCs intervention, generally MSCs in clinical studies for various diseases do not require human-leukocyte-antigens (HLA)-matching procedures. This also adds to their credit and therapeutic potential for the rapidly deteriorating COVID-19 context. Besides, some concerns regarding whether or not autologous MSCs from SARS-CoV-2 infected patients are infected with the virus, and whether the overall status of the infected-patient might affect these autologous-MSCs. Additionally, it’s noteworthy that MSCs count/function have been reported to decline with age (Pittenger et al., 2019), since the elderly are among the most vulnerable to suffer severe COVID-19, thus autologous option is absolutely questionable in those patients.

Again, this preference for allogenic transplantation raise a lot of issues regarding the use of MSCs as a “pharmaceutical product” or “stem cells drug.” The first issue is obtaining “clinical grade” MSCs of superior reproducible quality and reasonable cost (Golchin et al., 2020). Besides, factors such as timing of MSCs administration related to the severity of the case, especially with a disease such as COVID-19 pneumonia, frequency of dosing, number of cells and route of administration, either through injection or inhalation are all open questions that require further investigations. Especially that efficiency of MSCs therapeutics could be largely dependent on the route of delivery, as well as the transplantation regimen (Giri and Galipeau, 2020). We tackled these issues about WJ-MSCs potentials/challenges to treat diabetes in a review (Kamal and Kassem, 2020). We think that MSCs application in viral infections generally, and respiratory-tract viral infections particularly is a field that warrant further investigation, pre-clinical and clinical trials to sharpen this readily available weapon in preparation to upcoming pandemics like the one we are suffering right now.

It’s noteworthy here that given the non-invasive property of isolating MSCs from umbilical-cord, MSCs derived from UC-tissue (WJ-MSCs) have been particularly suggested as a potential option in these dangerous times for treating critically-ill COVID-19 patients under compassionate use protocols (Atluri et al., 2020). Importantly, we have to differentiate between the UC-tissue and the UC-blood (UCB). Each of these compartments provides different types of stem cells; MSCs are predominantly abundant in the UC-tissue/WJ, rather than UCB (Shetty et al., 2010).

Recently, few published reports shared some results from pilot studies which investigated the safety/efficacy of MSCs for COVID-19. Of these, a case report revealed the efficacy of WJ-MSCs infusion in a critically-ill COVID-19 patient. In that study, the authors reported that pulmonary functions showed significant improvement in 2 days, and the patient was discharged in 7 days after receiving cell-therapy (Zhang Y. et al., 2020).

In another study, a total of ten patients were enrolled; seven patients (1 critically-ill, 4 with severe symptoms, and 2 with mild symptoms) received MSCs-transplantation, while three patients (with severe symptoms) served as the control group and received placebo (Leng et al., 2020). After receiving MSCs intravenously, nearly all symptoms showed significant improvement within 2–4 days post-infusion, even the critically-ill elderly patient who received MSCs, showed an overall improvement and recovered. Moreover, pro-inflammatory cytokines showed dramatic reduction, this was associated with improvements in leukocytes-count, as well as increased levels of IL-10 and VEGF which promoted lung-repair. Furthermore, remarkable reduction in pneumonia infiltration was observed in chest computed-tomography scans, and patients did not suffer any side effects. On the other hand, regarding the placebo group, 1 patient died, and the other 2 remained suffering severe symptoms till the end of follow-up period. Moreover, the authors reported that MSCs expressed neither ACE2, nor TMPRSS2, which could provide them with natural immunity to SARS-CoV-2 (Leng et al., 2020). Despite the interesting findings, it’s important to remember that the study was indeed limited by the small patients’ cohort.

## Discussion

**FIGURE 1:**
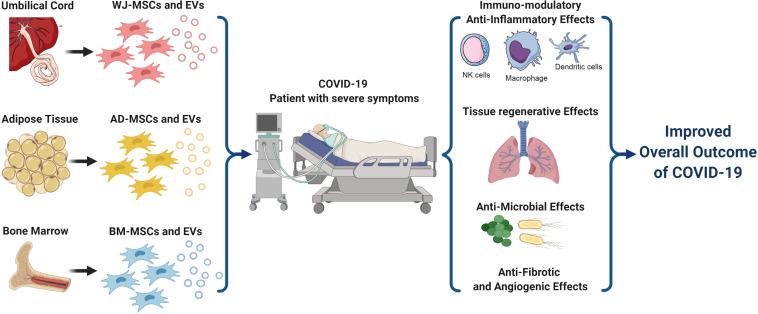
A schematic presentation for the different types of mesenchymal stem cells and their various therapeutic strategies implicated in COVID-19 treatment. MSCs and their derived EVs can efficiently intervene via several mechanisms to improve the overall outcome of COVID-19 especially in critically-ill patients with severe symptoms. These mechanisms include their immunomodulatory activities, tissue regenerative effects, anti-microbial effects, as well as their antifibrotic and angiogenic effects. AD, Adipose tissue; BM, Bone marrow; COVID-19, Corona Virus Disease-2019; EVs, Extra-Cellular vesicles; MSCs, Mesenchymal Stem Cells; and WJ, Wharton’s Jelly. Created by Biorender.com.

Knowing that severity of COVID-19 is mostly dependent on the patients’ own immune-response, since over-activation of the immune-system can cause a “cytokine storm” which in turn can cause ARDS, multi-organ damage, and ultimately may lead to death. Thus, harnessing the immunomodulatory properties of MSCs to ameliorate that cytokine-storm can indeed provide a golden key for treating severe cases of COVID-19. In fact, MSCs-transplantation can improve the overall outcome of COVID-19 via multiple strategies; (a)through their immunomodulatory effects, (b)via promoting tissue-repair and regeneration, (c)through their antifibrotic, as well as angiogenic and anti-microbial effects as summarized in Figure 1. All these mechanisms will interplay and intervene together to enhance lung-repair and protect various organs from any damage resulting from exaggerated immune-response.

Nevertheless, it’s important to keep in mind the concerns regarding suggested pro-coagulant effect of MSCs and/or their derived EVs (Silachev et al., 2019), especially for COVID-19 patients. Thus, for vulnerable patients, it’s important to keep an eye on patient coagulopathy and take proper thromboprophylactic measures while the clinical application of MSCs (Moll et al., 2020). Furthermore, in this regard, tissue factor (TF/CD142/coagulation factor III) has been highlighted as a major determinant of cell product hemocompatibility (Moll et al., 2019). TF/CD142 is a glycoprotein which triggers the coagulation cascade via inducing thrombin formation from pro-thrombin (Witkowski et al., 2016). Thus, MSCs which are going to be used for COVID-19 should express only low levels of TF/CD142, since the molecule by itself may initiate the clotting process, and also because MSCs from different sources have been reported to express varying degrees of TF/CD142 (Moll et al., 2019). It’s also important to remember the statement by the international society of extracellular-vesicles (ISEV) and international society for cell and gene therapy (ISCT) regarding the potential of MSCs-derived EVs as a treatment for COVID-19, and announcing that they do not currently endorse their use in COVID-19, without the generation of appropriate manufacturing and quality-control provisions, as well as pre-clinical safety and efficacy data, and proper regulatory oversight (Börger et al., 2020).

**FIGURE 2:**
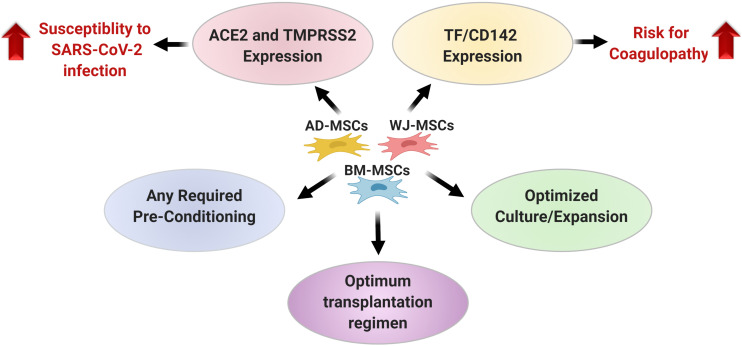
A summary for the critical issues that should be carefully considered when selecting a certain type of mesenchymal stem cells for COVID-19 treatment. AD, Adipose tissue; ACE2, Angiotensin converting enzyme; BM, Bone marrow; COVID-19, Corona Virus Disease-2019; MSCs, Mesenchymal Stem Cells; TF/CD142, Tissue factor; SARS-CoV-2, Severe acute respiratory syndrome corona virus-2; TMPRSS2, Transmembrane protease serine 2 and WJ, Wharton’s Jelly. Created by Biorender.com.

Conclusively, the published findings until now regarding therapeutic potential of MSCs for inflammatory-conditions generally, and for COVID-19 specifically are indeed encouraging. Nevertheless, several un-answered open-questions and critical issues should be carefully considered as summarized in Figure 2, and some of these issues remain as black boxes. These include whether pre-conditioning of MSCs will impact their beneficial therapeutic effects for COVID-19 or not. Accordingly, further research is warranted to elucidate how the process of MSCs-isolation and culture/expansion can be optimized in order to produce MSCs with the best therapeutic properties for COVID-19, not only in large amounts, but also in a reproducible manner. Such optimized approaches will help us to achieve tailored therapies for any condition generally, and for COVID-19 particularly, in a form of “personalized medicine” ultimately improving the cost-benefit returns (Pittenger et al., 2019).

Finally, MSCs derived from various sources were reported to have different properties in varying contexts. Thus, MSCs derived from which of these sources could provide the best therapeutic outcome for COVID-19 patients remains largely unknown. Future well-designed large-scale randomized-controlled trials comparing the therapeutic effects of MSCs derived from different sources will provide important insights in this regard. Similar studies are also warranted to compare the therapeutic benefit of a certain MSCs type, and its derived EVs.

## Author Contributions

Both authors collected the data and wrote the entire manuscript and they did not receive any form of sponsorship or honorarium for the material, and approved the final version of the manuscript.

## Conflict of Interest

The authors declare that the research was conducted in the absence of any commercial or financial relationships that could be construed as a potential conflict of interest.
